# Protocols for Personal Protective Equipment in a COVID-19 Medical Shelter

**DOI:** 10.1017/dmp.2020.244

**Published:** 2020-07-14

**Authors:** Sarah Hockaday, Kate Krause, Catherine Sobieski, Jeffrey N. Li, Rachel Hurst, Benjamin Ryan, Michael Leader, Dustin Smith, Ray Fowler, Andrew Tran, Stephen McMullan, Andrew Hogan, Paula Volk, Ronna Miller, B. Ward, Lindsay Flax, Ray Swienton

**Affiliations:** Department of Emergency Medicine, UT Southwestern Medical Center, Dallas, Texas; Department of Environmental Science, Baylor University, Waco, Texas; Naval Branch Health Clinic Jacksonville, Jacksonville, Florida; Naval Hospital Jacksonville, 2080 Child St, Jacksonville, Florida

**Keywords:** COVID 19, pandemics, disaster medicine, protective personal equipment, medical shelter

## Abstract

The coronavirus disease 2019 (COVID-19) has greatly impacted health-care systems worldwide, leading to an unprecedented rise in demand for health-care resources. In anticipation of an acute strain on established medical facilities in Dallas, Texas, federal officials worked in conjunction with local medical personnel to convert a convention center into a Federal Medical Station capable of caring for patients affected by COVID-19. A 200,000 square foot event space was designated as a direct patient care area, with surrounding spaces repurposed to house ancillary services. Given the highly transmissible nature of the novel coronavirus, the donning and doffing of personal protective equipment (PPE) was of particular importance for personnel staffing the facility. Furthermore, nationwide shortages in the availability of PPE necessitated the reuse of certain protective materials. This article seeks to delineate the procedures implemented regarding PPE in the setting of a COVID-19 disaster response shelter, including workspace flow, donning and doffing procedures, PPE conservation, and exposure event protocols.

The novel coronavirus severe acute respiratory syndrome coronavirus-2 (SARS-CoV-2) that causes coronavirus disease 2019 (COVID-19) emerged in Wuhan, China, in December 2019. Within a month, the virus had spread across the world, with outbreaks appearing in South Korea, Japan, and some locations in the United States (in Washington State).^1^ At the time of this publication, 5 months since its emergence, COVID-19 has been declared a pandemic responsible for more than 7.4 million cases and 417,000 deaths across 188 countries.^2^

The rapid spread of COVID-19 has greatly impacted the United States, depleted the national stockpile of personal protective equipment (PPE), and led to controlled allocation of health-care resources in hospitals across the country. Early COVID-19 predictability modeling by the White House Task Force and the US Department of Health and Human Services identified Dallas County among areas at significant risk of exceeding the local hospitals’ services infrastructure. The region of concern included Dallas County and surrounding areas, which comprises the ninth highest population density within the United States.^3^

In an effort to mitigate the predicted surge in COVID-19 cases within the Dallas area, joint federal, state, and local agencies converted a 200,000 square foot convention center space into a pandemic medical shelter, retrofitted for pathogen containment and observation of 250 patients affected by COVID-19. This article details the PPE policies and procedures implemented to protect shelter personnel from this highly contagious respiratory pathogen.

## DESCRIPTION OF FACILITY

**FIGURE 1:**
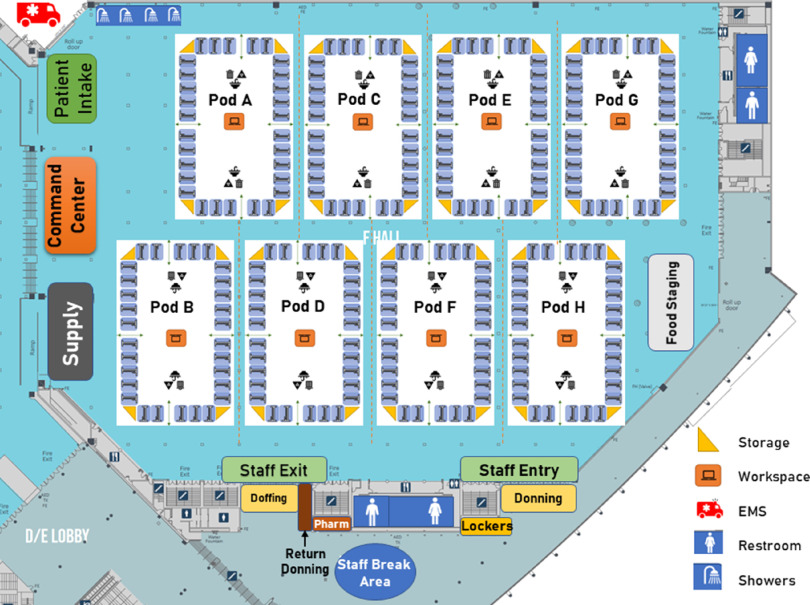
Federal Medical Station layout, Kay B. Hutchison Convention Center, Hall F.

The Federal Medical Station was designed to house 250 beds staffed by an assigned Naval Expeditionary Medical Unit. Beds were sectioned into 8 pods with 30-32 beds each, all separated by partitions (Figure 1). Each pod was to be managed by a nurse and 6 Navy hospital corpsmen. Medical providers were to be stationed at the patient intake area to receive patients from ambulances. All boundaries of this convention exhibition space, Hall F, were monitored by personnel and separated from adjacent areas by solid barriers. Designated areas for doffing and donning of PPE are described in the following diagrams.

Diagram created by Hockaday et al.

**FIGURE 2:**
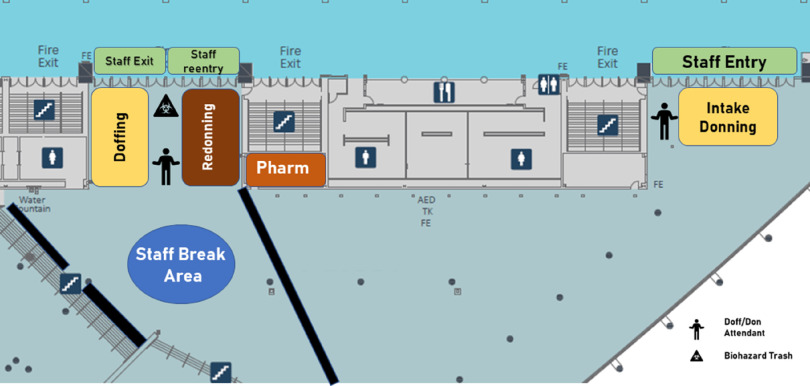
Patient Care Area, Entry and Exit Points.

The section of the convention space detailed in Figure 2 was designated as a retrofitted area for donning and doffing of PPE. Preexisting fire exits and restrooms provided a barrier between the 2 points of entry. Three tents were to be constructed: 1 tent at the first entrance (intake donning) and 2 tents at the second entrance (doffing and re-donning). Trained attendants were to be stationed in each tent to assist with donning, redonning, and doffing.

Diagram created by Hockaday et al.

Due to the unique nature of a special pathogen shelter, it was necessary to physically separate the intake donning space from the patient care area. At the intake donning tent, all personnel would don fresh PPE before entrance into the patient care area. The second entryway, with tents designated for doffing and redonning, was to be positioned near a staff respite area. This area would serve as a singular exit point, for personnel exiting the shelter and those taking breaks. Staff planning to re-enter the patient care area during the same shift would conserve their PPE (as described below) to don again in the redonning area under the direction of a trained attendant.

**FIGURE 3:**
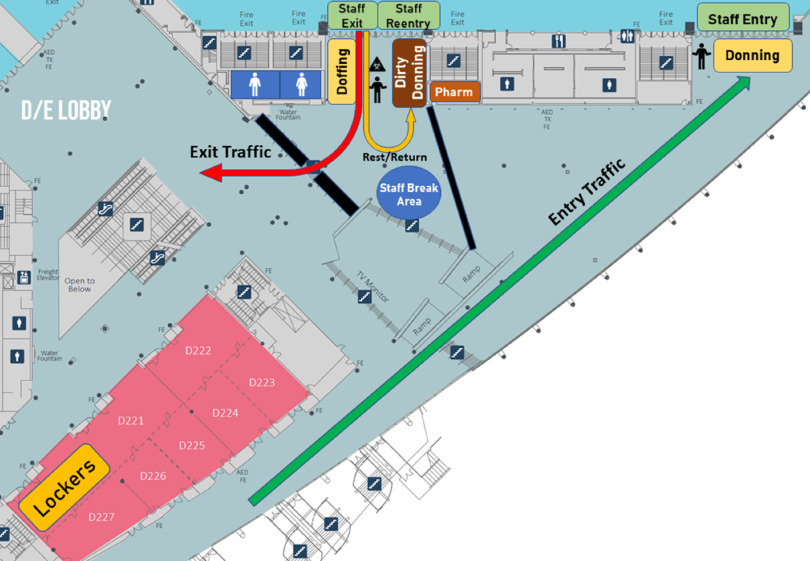
Traffic Flow Schematic for Entering and Exiting Patient Care Area.

To further ensure the safety of all personnel, a traffic flow pattern was established as detailed in Figure 3. All staff were to enter the facility by means of the designated “entry traffic” path to the intake donning tent. The exit route for staff leaving the shelter is labeled “exit traffic.” These pathways were designed to funnel personnel through separate hallways to minimize risk of pathogen transmission.

Diagram created by Hockaday et al.

## PATIENT CARE AREA PERSONNEL AND PROTECTIVE EQUIPMENT

PPE is defined by the Occupational Safety and Health Administration (OSHA) as “specialized clothing or equipment worn to minimize exposure to hazards.” The PPE for this environment was chosen to provide contact and droplet precautions for workers in the patient care area, in compliance with OSHA’s Personal Protective Equipment standard (29 CFR 1910.132).^4^

The following PPE was to be donned by all personnel entering the patient care area: disposable gown, fit-tested particulate respirator (N95 mask), full face shield, gloves, shoe covers, and surgical masks.

Personnel approved for entry into the patient care area were predetermined by the Medical Operations team to be essential to daily operations. Personnel with such designation included patient care staff (physician, nurses, hospital corpsmen), security (police officers, security guards), and facilities management (environmental service workers, food service workers).

PPE donning and doffing are crucial for infection prevention and maintaining the health of the workforce but can be intimidating and complex to those not familiar with the procedures. Thus, when creating the Federal Medical Station, staff from all levels were recruited to actively participate in the planning, development, and implementation of the protocols for PPE. Emory University’s guidelines served as the basis for the PPE donning and doffing protocols.^5^ Multiple modifications were made after testing to accommodate the specific needs of staff, the available PPE, and to better clarify procedures. To ensure compliance with PPE protocols, plans were developed to have trained PPE attendants at all donning/doffing stations to assist with PPE procedures and to ensure the safety of personnel working within the shelter.

## PPE CONSERVATION

The current COVID-19 pandemic has placed an unprecedented burden on medical resources within the United States.^6^ The increased demand for PPE has created supply shortages across the country, hampering the ability of health-care systems to conduct patient care under current standing PPE protocols. Health and Human Services judiciously allocated PPE to areas most affected by the pandemic at the state and federal levels; however, demand continues to exceed the national supply.^6^ Thus, when planning the Federal Medical Station, it was necessary to put into action proactive measures to conserve PPE. The protocols defined methods for repeat donning of cleaned face shields and reused N95 masks based on Emory University Conservation of PPE guidelines and the Defense Health Agency (DHA) Infection Prevention Update for the COVID19 pandemic.^5,7^ To maximize conservation efforts, close inventory of PPE and use of “burn rate” calculators to estimate resupply needs were incorporated into the protocol.^8^

## PPE DISPOSAL

All PPE worn within the medical shelter was to be properly discarded into biohazard containers within the shelter and at doffing stations. Disposal of such medical waste was planned to be performed by environmental service workers who were managed by a contracted waste management company according to state and local laws.

## DONNING AND DOFFING PROCEDURES

Guidelines are detailed below to describe donning and doffing procedures in support of PPE conservation. These guidelines are subject to revision based on the unique circumstances arising during operations. Gloves were to be changed between each patient encounter. For personnel temporarily exiting the patient care area with the intention of reentering, N95 masks were to be conserved and stored in labeled paper bags. It was planned to have face shields cleaned and placed in a designated “clean” zone of the redonning area to dry. N95 masks were to be reused a maximum of 5 times during one 12-h shift. Trained observers stationed at PPE tents were to ensure compliance with PPE protocols and the safety of the staff. The protocols regarding PPE donning and doffing are detailed below.

INTAKE DONNING

Personal items:

1. Ensure all jewelry (wedding bands, watches) have been stored in the locker area
2. Thoroughly disinfect hands with hand sanitizer for at least 20 s

Gown and gloves:

1. Put on gown by taking care to put thumbs through thumb loops, then placing head through opening at the top of gown. Tie strings around waist in front, if possible (can be tied at back if necessary)
2. Put gloves on, pulling glove over sleeve of gown

Mask:

1. Put on mask:
  - If **medical staff**, put on N95 by the bottom strap and then the top strap. Ensure that straps do not cross. Pinch nose of N95 mask to create seal to face. Place gloved hands over front of N95 respirator and take a deep breath in, then exhale. If air is felt escaping from sides of mask, tighten straps, readjust nose of mask, and repeat.
  - If **nonmedical staff**, put on surgical mask by placing loops around ears, taking care not to touch face. Fit mask to bridge of nose.
2. Sanitize gloves with hand sanitizer for at least 20 s

Face shield:

1. Put on face shield by pulling strap far overhead, taking care not to touch the face
2. Sanitize gloves with hand sanitizer for at least 20 s

Shoe covers:

1. Sit down in chair and place shoe covers over shoes
2. Sanitize gloves with hand sanitizer for at least 20 s
3. Enter patient care area

Adapted from guidelines published by Emory University and the DHA Infection Prevention Update.^5,7^

**Table:** 

**BETWEEN-PATIENT DOFFING** 1. Sanitize gloves with hand sanitizer for at least 20 s 2. Break gown waist tie 3. Cross arms and grasp gown at front of shoulders 4. Lean forward at the waist and pull gown down and away from the body in controlled manner so that the ties break at the nape of the neck 5. While removing gown, roll the gown inside out into a bundle toward hands 6. As you are removing the gown, peel off your gloves 7. Discard gown and gloves 8. Sanitize hands with hand sanitizer for at least 20 s Adapted from guidelines published by Emory University and the DHA Infection Prevention Update.^5,7^	**BETWEEN-PATIENT DONNING** 1. Sanitize hands with hand sanitizer for at least 20 s 2. Open gown and carefully unfold 3. Cut the posterior midline of the nape of gown with scissors 4. Don arms of gown, and put thumbs through thumb loops 5. Tie loose ends of nape of gown behind the neck, taking care not to touch face shield 6. Sanitize hands with hand sanitizer for at least 20 s 7. Put gloves on, pulling glove over sleeve of gown Adapted from guidelines published by Emory University and the DHA Infection Prevention Update.^5,7^

**Table:** 

**DOFFING FOR PPE CONSERVATION**Gown and gloves: 1. Sanitize glove with hand sanitizer for at least 20 s 2. Break gown waist tie 3. Cross arms and grasp gown at front of shoulders 4. Lean forward at the waist and pull gown down and away from the body in controlled manner so that the ties break at the nape of the neck 5. While removing gown, roll the gown inside out into a bundle toward hands 6. As you are removing the gown, peel off your gloves 7. Discard gown and gloves 8. Sanitize hands with hand sanitizer for at least 20 s Face shield: 9. Don fresh gloves 10. Place sanitizing wipe flat on table to create a clean surface 11. Remove face shield from the back by lifting head strap overhead and place on wipe 12. If wearing glasses, remove now and place on wipe 13. Sanitize gloves with hand sanitizer for at least 20 s 14. Obtain a new sanitizing wipe and clean the face shield, starting with the front and back, then the elastic band, then the foam band 15. Place face shield standing vertically with foam side down to dry; face shield must remain visibly wet for 3 min 16. Sanitize gloves with hand sanitizer for at least 20 s Mask: 17. Remove mask: • If **medical staff**, use 1 hand to stabilize N95 mask while removing straps. Pull bottom strap over head; repeat with top strap. Take care to avoid touching face with either hands or straps. Place mask face-down inside paper storage bag and avoid touching sides of bag. • If **nonmedical staff**, pinch elastic straps of surgical mask between fingers and pull straps out and forward. Pull mask far away from body and discard. 18. Sanitize gloves with hand sanitizer for at least 20 s Shoe covers: 19. Sit down in chair to remove shoe covers 20. With gloved hands, grasp back of shoe cover at heel and pull back to remove heel from cover then pull cover down toward toes 21. Repeat process on other foot taking care not to touch bare skin with hands 22. Discard shoe covers 23. Sanitize gloves with hand sanitizer for at least 20 s 24. Remove and discard gloves 25. Wash hands and forearms up to the elbow with soap and water for at least 20 s Adapted from guidelines published by Emory University and the DHA Infection Prevention Update.^5,7^	**DOFFING FOR END OF SHIFT**Gown and gloves: 1. Sanitize glove with hand sanitizer for at least 20 s 2. Break gown waist tie 3. Cross arms and grasp gown at front of shoulders 4. Lean forward at the waist and pull gown down and away from the body in controlled manner so that the ties break at the nape of the neck 5. While removing gown, roll the gown inside out into a bundle toward hands 6. As you are removing the gown, peel off your gloves 7. Discard gown and gloves 8. Sanitize hands with hand sanitizer for at least 20 s Face shield: 9. Don fresh gloves 10. Remove face shield from the back by lifting head strap overhead and discard 11. Sanitize gloves with hand sanitizer for at least 20 s Mask: 12. Remove mask: • If **medical staff**, use 1 hand to stabilize N95 mask while removing straps. Pull bottom strap over head; repeat with top strap. Take care to avoid touching face with either hands or straps. Discard N95. • If **nonmedical staff**, pinch elastic straps of surgical mask between fingers and pull straps out and forward. Pull mask far away from body and discard. 13. Sanitize glove with hand sanitizer for at least 20 s Shoe covers: 14. Sit down in chair to remove shoe covers 15. With gloved hands, grasp back of shoe cover at heel and pull back to remove heel from cover then pull cover down toward toe 16. Repeat process on other foot taking care not to touch bare skin with hands 17. Discard shoe covers 18. Remove and discard gloves 19. Wash hands and forearms up to the elbow with soap and water for at least 20 s Adapted from guidelines published by Emory University and the DHA Infection Prevention Update.^5,7^

REDONNING

Gown and gloves:

1. Put on gown by placing arms into each sleeve, putting thumbs through thumb loops, then placing head through opening at top of gown. Tie strings around waist in front, if possible (can be tied at back if necessary)
2. Put on gloves, pulling glove over sleeve of gown

Mask:

1. Put on mask:
  - If **medical staff**, place sanitizing wipe flat on table to create a clean surface. Retrieve N95 from storage bag, taking care to avoid touching bag, and place on wipe. Sanitize gloves with hand sanitizer for at least 20 s. Firmly grasp N95 respirator with nondominant hand. Using dominant hand, place the bottom strap and then the top strap, pulling far over head. Ensure that straps do not cross, and check seal.
  - If **nonmedical staff**, put on surgical mask by placing loops around ears, taking care not to touch face. Fit mask to bridge of nose.
2. Sanitize gloves with hand sanitizer for at least 20 s

Face shield:

1. Retrieve and don face shield by pulling strap far overhead, taking care not to touch the face
2. Sanitize gloves with hand sanitizer for at least 20 s

Shoe covers:

1. Sit down in chair and place shoe covers over shoes
2. Sanitize gloves with hand sanitizer for at least 20 s
3. Enter patient care area

Adapted from guidelines published by Emory University and the DHA Infection Prevention Update.^5,7^

## PPE EXPOSURES

Policies were delineated for facility personnel whose PPE was contaminated, damaged, or inadvertently removed while in the patient care area. In accordance with DHA guidelines, PPE contaminated with blood, respiratory secretions, or other bodily fluids was disposed of and replaced.^7^ Due to the continued exposure risk while navigating the patient care area, staff members were instructed to continue wearing the damaged or contaminated PPE while exiting toward the doffing station. At that point, the damaged item was discarded and a new protective item donned under the supervision of a trained PPE attendant.

In scenarios involving removal of masks in the patient care area, the affected person should immediately mobilize toward the doffing zone exit, maintaining a minimum distance of 6 ft from all staff and patients. The affected person was then considered at high risk for exposure and would undergo self-quarantine for 14 d.

## CONCLUSIONS

Prior coronavirus outbreaks (Middle East respiratory syndrome coronavirus [MERS-CoV], 2013; SARS-CoV, 2002) raised questions of how new strains of this virus will affect future generations.^9^ The current pandemic demonstrates our continued susceptibility to novel infectious diseases and has revealed shortcomings of health-care infrastructure across all continents. Deficits in PPE and limited pathogen containment have been described within communities in the United States (eg, New York City).^6^ In anticipation of such challenges in the Dallas area, it was our goal to create a patient care facility that used methods of PPE conservation, while attempting to maximize staff safety by using defined protocols. At this time, no current research has validated the safety of reused/recycled PPE, yet the shortage has necessitated alternative approaches.^6^ Further research involving alternate forms of effective PPE conservation methods while studying medical staff infection rates is needed and will no doubt have a positive impact on protection of both patient and provider health during future pandemics. Analysis of operations in pandemic medical shelters, such as the one described, can be performed to improve safety procedures for future events.

## Copyright Disclaimer

I am a military service member or federal/contracted employee of the United States government. This work was prepared as part of my official duties. Title 17 U.S.C. 105 provides that copyright protection under this title is not available for any work of the United States Government. Title 17 U.S.C. 101 defines a US Government work as work prepared by a military service member or employee of the US Government as part of that person’s official duties.
